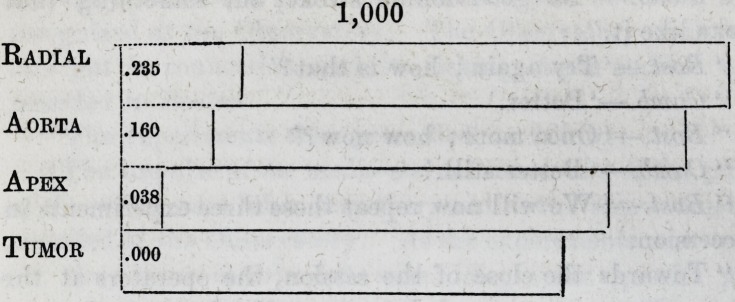# Remarkable Malformation.—Notes of Observations upon M. E. A. Groux, with Congenital Fissure of the Sternum

**Published:** 1859-04

**Authors:** A. Snowden Piggot

**Affiliations:** Professor of Anatomy in Baltimore College of Dental Surgery.


*Remarkable Malformation.—Notes of Observations upon M. E. A. Groux, with Congenital Fissure of the Sternum.* By A. Snowden Piggot, M. D., Professor of Anatomy in Baltimore College of Dental Surgery.

On Tuesday evening, February 1st, Mr. Eugene A. Groux, whose remarkable malformation of the sternum has attracted so much attention in Europe, exhibited himself before the class at the Baltimore College of Dental Surgery. The phenomena of his condition are so remarkable and instructive that I have thought a report of them might prove generally interesting to the readers of this Journal.

Before stating the present condition of the patient, I shall lay before the reader a sketch of his history, which has been so succinctly related by Dr. Upham, of Boston, that I cannot do better than quote his language from the records of the Society for Medical Improvement in that city, as published in the Boston Medical and Surgical Journal.

"M. Eugene A. Groux was born in Hamburg on the 10th of January, 1831. His father was also a native of Hamburg ; his mother, of Bordeaux, in France. The congenital fissure of the sternum, which forms his anatomical peculiarity, though known, of course, at birth, does not seem to have excited any marked attention. There is a tradition of a consultation being held as to whether a swathe or bandage should not be applied to his chest, to bring the divided sternum into closer apposition. Fortunately for himself, and for science, perhaps, he was left for nature to treat. At the age of about three years, he was subjected to examination in a private medical society at Hamburg, of which he thinks he has some recollection still. Being otherwise of feeble and delicate organization, when five years old he was placed at school in the country, where he remained till the age of 12. He then returned to his native city, and joined a school of higher grade for some years more. When 15 years of age, he went to London, and was employed as an assistant in business with his uncle, a soap-boiler by trade. In this capacity he remained for a period of a year and six months, when he was seized with a severe attack of cholera, which was then epidemic in England. In this illness he was attended by Dr. Beneke, physician to a German hospital in London, to whom he communicated the fact of his singular malformation. Dr. B. was a man to understand and appreciate the value of the case. On his recovery, through the instrumentality of Dr. B. he was presented to most of the eminent physicians and surgeons of London, and various reports of his case were at that time published in the English journals. He remained in London some ten or twelve weeks after this attack, when he returned to Hamburg, and entered a well-known mercantile house in that city. This is an establishment corresponding with the wholesale drug and medicine stores with us. His duty was here, among other things, to assist in the analyzation of drugs, which led to more or less familiarity with chemistry and pharmacy. In this employment he remained for two years more or less, when he was attacked with pleurisy of the right side, accompanied by an inconsiderable amount of hæmoptysis. He attributes this attack to breathing the fumes of phosphorus. He was thus compelled to leave the establishment, and, finding himself, after a somewhat tedious convalescence, in a feeble state, remained about home, employing his time, by the advice of his physicians, in out-door exercises and sports.

"It was now, being advised to travel for the benefit of his health, that he formed the determination to exhibit his case to the medical world. He therefore set out on his tour in the latter part of the year 1851, since which time he has visited nearly all the large towns and cities in Europe—has been presented to the various medical and learned societies, and has also subjected himself for private examination to numberless physicians and medical men of eminence in Germany, Holland, Sweden, Russia, Spain, France and the kingdom of Great Britain.

"After traveling a couple of years, he resolved to enter systematically on the study of anatomy and physiology; and for this purpose connected himself with the schools and hospitals at Vienna, and subsequently in Paris, where he shared as a student, the facilities so abundantly afforded in those capitals. In 1856, he remained for about six months in Russia, whither he went at the call of the Government, preferred through Dr. Zdekauer, his time being there divided in the cities of Dorpat, St. Petersburg and Moscow. Some four or five weeks ago he arrived in New York, with the purpose of making the tour of this country.

"The case of M. Groux, it would appear, is, in most respects, unique. Two cases, however, are on record, which approach in some degree, the peculiar malformation we have before us. The first is that detailed by Harvey of a young nobleman—the son of Lord Montgomery—in whom there existed a cavity of the left side (the consequence of a fall, attended with fracture of the ribs, and followed by a suppurative abscess) through which the heart could both be seen and touched; the second, that mentioned by Mr. Lyons, of Dublin, as having occurred in a boy 14 years of age, laboring under a deformity consisting of lateral curvature of the spine and a deviation of the ribs, admitting, through a triangular space (covered only by integument) thus left in the side of his chest, a partial view of the motions of the heart. And I beg leave to read from the manuscript of Dr. J. Hughes Bennet, of Edinburg, the record of a case still more analogous, with which I will close this imperfect sketch. 'Eleven or twelve years ago, when Pathologist to the Royal Infirmary, I found in the body of a woman who died under the care of Dr. W. Robertson, the very same malformation of the sternum which is to be found in M. Groux. Dr. R. informs me that he has no notes of the case, and he does not remember having made any particular observation upon it. The specimen is preserved in the Museum of this University, and I have always regarded it as an unique preparation. M. Groux informs me it is the only one of the kind which, in the course of his visits to the various medical schools, he has met with.' It does not appear, however, that any observations of this case were made during life."

The case is a specimen of an exceedingly rare malformation, of which the instance named in Dr. Upham's sketch, is the only one I have seen recorded. My friend, Dr. Chas. Frick, Professor of Therapeutics in the University of Maryland, tells me that he witnessed such an one at the Baltimore Almshouse, while he was pursuing his studies at that institution. He preserved the bone, but has no recollection of any special phenomena connected with the malformation, occurring during life.

The condition of things in Mr. Groux's chest is a true fissure, not a total absence of the sternum. The osseous formation, as far as observation through the skin can determine it, appears to be confined to a narrow bony belt, occupying the position of the costal margins of the normally formed bone. To this belt, the ribs and clavicles are attached. Of course, therefore, there is a total deficiency of bone from the neck down to the neighborhood of the ensiform cartilage, in the middle line of the front of the chest. The space thus left contains no cartilage, but appears to be closed with elastic fibrous tissue, covered with the common integument.

When examined during tranquil respiration, the anterior portion of the chest, in the middle line, presents a depression or groove, shaped like an elongated V. The width of this depression is about an inch and a quarter between the clavicles, gradually narrowing to its apex, which is near the ensiform cartilage. The depth averages a half inch, being slightly increased during inspiration, and diminished during expiration. Muscular action and violent respiratory movements greatly change these conditions. The former is capable of increasing its width to double that described, while the latter can either greatly deepen the fissure, or produce a bulging instead of a depression, as we shall presently see.

During ordinary tranquil respiration, a single pulsation is visible in the upper part of the fissure, at about the level of the fourth rib, but, upon a deeper inspiration, two are visible, the second being seen over the apex of the heart towards the bottom of the fissure.

The phenomena presented by this malformation may be described under three heads, as they relate to the muscular action of the upper extremity, to respiratory movements proper, and to the phenomena of the circulation.

*Muscular Action.*—The width of the fissure is very much under the control of the patient. When the hands are clasped so that the humeri are fixed, contraction of the pectorals greatly expands the fissure, separating the bony edges to the extent of two and a half inches. A moment's consideration of the anatomical position of these muscles, sufficiently explains this action. The ordinary fixed points of these muscles are the ribs of either side, and their contraction, of course, draws the arms towards the chest. But when the arms are fixed, then the tendency of this contraction must be to draw the sides of the chest towards the arms. This, which is impossible with a naturally formed sternum is effected in M. Groux's case, thus accomplishing the separation of the sides of the fissure. It is to be remarked, that upon raising the clasped hands, the muscles are thrown out of action and the fissure collapses.

The action of the deltoids is just the reverse of this. When the hands are clasped, as before, and these muscles brought into action, the fissure is closed, one of the sides overriding the other. This depends upon an approximation of the clavicle and humerus consequent upon the contraction of the deltoid. The humerus being the fixed point, contraction of the deltoid must lower the point of the shoulder, depressing both clavicle and scapula. Now, owing to the mode of union of the clavicle with the sternum, this bone becomes, under such circumstances, a lever, pressing upon the sterno-clavicular articulation, forcing it inwards from either side, and so closing the fissure.

*Respiratory Movements.*—During ordinary respiration, a change is observed in the appearance of the fissure, according as the muscles of expiration or of inspiration are used. This change is far more manifest when these actions are more violent. A deep inspiration greatly deepens the fissure, and this effect is much more marked when the inspiratory motions are made at the same time that the mouth and nostrils are closed. The experiment well illustrates the passive condition of the lungs during inspiration, and the dependence of that function upon atmospheric pressure.

Expiration forces out a tumor which greatly distends the integument over the upper part of the fissure. Percussion upon this, gives a clear sound, showing that it is the lung which is thus forced out through the bony parietes of the chest. This is particularly marked during the violent efforts of coughing, when the lung suddenly springs up, forming this large tumor, and as speedily falls back again out of sight. This remarkable and interesting phenomenon is not likely to be seen in another person during the present generation.

Connected with these respiratory acts, is an influence upon the pulse which attracted no liitle attention. By taking a deep inspiration, the arms hanging down, M. Groux is able to suspend the pulse in the upper extremities. This appears to be accomplished by the pressure of the distended lungs upon the first rib, lifting it until the subclavian arteries are compressed between it and the clavicle. This is not so uncommon an effect as it was at first supposed. The students amused themselves with experiments upon their own persons, and one of them, who probably had an unusually movable first rib, called my attention to the fact that he was able to accomplish the same feat. A letter to the editors of the Boston Medical and Surgical Journal for January 20th, gives an account of a consumptive patient of Dr. Sibson's, at St. Mary's Hospital, London, who possessed the same power over the arteries of the upper extremity.

*Phenomena of the Circulation.*—There has been a very remarkable diversity of opinion among men of eminence in the medical profession in reference to the cause of the pulsation visible at the upper part of the fissure. It consists of an oval tumor, about the size of a large walnut, which rises from below upwards, and towards the left, and swiftly disappears, with a quick, undulatory movement, in the opposite direction. These two motions constitute the pulsation in question. At a point somewhat higher, another pulsation can be felt, and at the lower portion of the fissure, towards the left, is seen and felt the throb of the apex of the heart.

The determination of the synchronism, or the contrary, of the movements in question, cannot be made evident to the touch, as that sense does not act quickly enough. The eye must appreciate it. To render this visible, M. Groux, whose interest in his condition and anxiety fully to expound it cannot be too highly commended, has provided himself with a pair of sphygmoscopes. The sphygmoscope, as our readers are probably aware, consists of a bell-shaped glass, covered water-tight with India rubber, which extends above it in a long flexible tube. To the free end of this flexible tube is attached a glass tube, filled with some colored liquid, which by its rise and fall, registers the motions of the parts over which the bell glass rests. M. Groux's instruments are filled with different colored liquids, one blue, the other red, so that the difference between the pulsations can be more readily seen.

On planting these, one over the apex of the heart, and the other over the pulsating tumor, it was evident that the movements were not synchronous, while those of the two ventricles were perfectly so. It is quite clear, therefore, that this pulsation cannot be that of the *infundibum* or *conus arteriosus*,* as has been supposed by some. Two other opinions have been advanced, one that the pulsation in question is caused by the right auricle, the other that it is the aorta itself which is seen and felt.

The question which has been so much discussed, has been at last finally settled by a series of very delicate experiments, performed with great skill and perseverance, by Dr. J. B. Upham, of Boston. Dissatisfied with the appliances for the determination of this vexed question, Dr. U. proceeded to contrive more efficient apparatus. His first idea was to render the sounds audible by an apparatus to which he gave the name of *sphygmosphone*. Its first form was nearly that of a sphygmoscope, with a float on the top of the fluid, which as it rose, gave an impulse upon a sonorous body placed above it. This, however, was abandoned for the more delicate arrangement of an electro-magnetic appa-

* The *infundibum* or *conus arteriosus* is the dilatation of the right ventricle at the origin of the pulmonary artery.

ratus, with a circuit-breaker attached to the sphgymosphone. In this manner sounds were obtained, and the asynchronism of the pulse of the tumor and that of the apex of the heart demonstrated.

The rest of the history of these experiments is so well told by Dr. Upham, in his interesting paper in the Boston Medical and Surgical Joural, that we will allow him to speak for himself.

"At the next session, we found ourselves in condition to obtain and to note satisfactory results. And our first design being to ascertain beyond question whether the impulse of the prominent pulsating tumor, in the middle of the sternal fissure, is or is not synchronous with that of the apex of the heart, we made use of an instrument called the 'Telegraphic Repeater,' * which is so constructed that of any two motions, that which is first, by ever so brief an interval, moves its armature and produces its sound, to the entire exclusion of the other. It mathematically follows that, if the two communicated motions are *synchronous*, neither armature will move; this, however, presupposes a high degree of perfection in the mechanism. Suffice it to say, that, with this apparatus, the instruments being applied to the medio-sternal tumor and to the apex, it was the impulse from the first which invariably set in motion the corresponding armature and gave out its sound.

"In our subsequent sessions, the 'Repeater' was set aside, and a 'Morse's double register' used in its place. This was so adjusted as to give forth two sounds, differing in pitch, and at the same time record the motions on paper, in the same way that ordinary telegraphic communications are written. Then, by the intervention of the electric clock, which was also made to mark its seconds on paper, it was easy to measure the time of the pulse-beats themselves, as well as the interval in the pulsation of any two points in the round of the circulation.

* This is an instrument used in telegraphing *through* messages over long lines, It is the joint invention of Mr. Farmer and the late Mr. A. F. Woodman, of Portland.

"Not to go, at this time, too minutely and tediously into description, I will here give the result by calculation of a few of these trials, including some witnessed by gentlemen present on the evening of the 5th of January inst., and afterward repeated in connection with the delicate chronographic apparatus in the Observatory at Cambridge. Before doing this, however, let me briefly allude to the Cambridge experiments, since they were in their nature, it is believed, both novel and interesting. They were done in the afternoon and evening of the 8th of January, Mr. Bond having, in the kindest manner, placed his beautiful apparatus in the Observatory at our disposal. Our forces were, on this occasion, divided—Mr. Groux, Mr. Farmer, Mr. Rogers and myself taking our position in the private apartment of the City Telegraph rooms in Court Square; and Mr. Stearns, the present able and efficient Superintendent of the Boston Fire-Alarm System, accompanied by Mr. Rennard, recently of the Fire-Alarm Office, St. Louis, going over to the Observatory. The telegraph between the central office in Boston and the Observatory, let me add, was also kindly placed at our disposal—and, furthermore, I will say that the instruments used here were furnished from the City Fire-Alarm Office, and were the best of their kind.

"At half past 3, P. M., a telegraphic notice from the Observatory signified that everything was in readiness there. But from the exhaustion and great nervous agitation of M. Groux, consequent upon recent illness, it was impossible to commence immediately the regular series of experiments, and nearly a couple of hours were spent in preliminary trials and tests. The line being found in perfect working order, the experimental apparatus at both ends also working beautifully, and Mr. Groux being now in a condition of comparative quiet, operations were commenced in earnest at about half past 5 o'clock. Some extracts from the original records, taken down in Boston and Cambridge simultaneously, will perhaps more graphically portray the nature of our proceedings.

"To begin—the beat of the pulsating tumor in the mediosternal space was tried. We were able to get several consecutive beats, which were also duly recognized at the Observatory. Next, a series of apex-beats was obtained, and recognized at the Observatory. The Observatory clock was now put in connection, and its tickings made audible and recorded in Boston.

"The experiments then proceeded, as follows:

"The impulse of the medio-sternal tumor and of the pulse at the wrist were taken together, and at the same moment recorded at the Observatory. As the experiments now went on, they were interlarded with telegraphic queries and answers; and for the sake of clearness, we will prefix. when necessary, the words *Boston* and *Cambridge* to the parts of this dialogue, according as they emanated from the one place or the other.

"After the experiment just alluded to, information was conveyed that it would be repeated.

"*Cambridge.*—'Aye, aye.'

"*Boston.*—'Good signals these, save them.'

"*Camb.*—'Shall we put in the clock now?'

"*Bost.*—'Yes. And as our next experiment, we will try the apex and wrist.'

"*Camb.*—'Go ahead.'

"*Bost.*—'Any good signals then?'

"*Camb.*—'Yes, one or two.'

"*Bost.*—'We will try that again. Any of these good?'

"*Camb.*—'Some of them very good.'

"*Bost.*—'About what difference in time between the beats in this experiment?'

"*Camb.*—'About two-tenths of a second.'

"*Bost.*—'In which does the difference appear greatest, this or the preceding experiment?'

"*Camb.*—'Should say the former.'

"This question being repeated after additional trials, the reply was, 'Wait till we can calculate them;' and, shortly afterward, an answer was received, 'The *former*, by a minute interval.'

"*Bost.*—'Now we will pass to another experiment.* Do you get a single or double stroke?'

"*Camb.*—'No good double stroke, but something that looks like it.'

"*Bost.*—'Try again; how is that?'

"*Camb.*—'Better.'

"*Bost.*—'Once more; how now?'

"*Camb.*—'Better still.'

"*Bost.*—'We will now repeat these three experiments in succession.'

"Towards the close of the session, the operators at the Observatory were requested to count the beats to be sent over during the space of one minute. I then applied the instrument to the radial artery at my own wrist, an assistant taking the pulse at the other wrist. It was ascertained by counting to be sixty-six in the minute. The question was now put to Cambridge, 'How many?'

"*Camb.*—'Sixty-six.'

"*Bost.*—'Once again.'

"Mr. Groux now applied the instrument to the medio-sternal tumor, for the period of a minute, and its pulsations were found to be seventy-two. The query was again put, 'How many?'

"*Camb.*—'Seventy-two.'

"But the above will suffice to show the nature of our proceedings; this session was continued without intermission for six hours.

"The following are some of the important results obtained which bear upon the question at issue: the whole number of sessions thus far has been ten—the calculations (made by Mr. Farmer) are based on the average of selected ex-

* The operators at the Observatory were not informed previously of the nature of this experiment. It was an attempt to record the medio-sternal and apex beats, by applying the sphygmosphones to these points direct—an exceedingly delicate test, tried repeatedly with success in our private experiments. Prior to the response from Cambridge, Mr. Farmer remarked, that with a single line of communication only, it would be impossible to note clearly so minute a double beat at the Observatory.

amples taken from all the experiments. They are expressed, in a rude way, by the diagram below:

and may be thus stated. The whole duration of the pulse-beat is represented by 1,000. Then the commencement of the beat proceeding from the medio-sternal tumor being .000, the interval to the apex-beat was found to be .038; to that of the ascending aorta, at its junction with the arch, .160; that of the radial artery at the wrist, .235; being in thousandths of a pulse-beat.*

"Lastly, when at the final session (on the day preceding M. Groux's departure to Philadelphia) the ends of both the instruments were placed, as nearly as possible, over the apex of the heart, the result, both to the ear, and as recorded by the chronograph, was absolutely a synchronism of sounds. Calculations were also made as to the time in which the heart's impulse is transmitted to the carotids, the temporal arteries, the abdominal aorta, and other points in the circulation, which, with other experiments, may be given at some future time."

It has thus been reserved for our own country to settle these vexed questions. It is evident that the pulsating tumor in the upper part of the fissure is the auricle.

*** Taking the Cambridge experiments alone, and the above intervals would be expressed by the figures .854, .156 and .237 respectively.**

## Figures and Tables

**Figure f1:**